# Rhabdomyolysis and Hepatitis Associated with Oropouche Fever: Brazil

**DOI:** 10.4269/ajtmh.25-0107

**Published:** 2025-08-14

**Authors:** Kalyne Gabrielle Caetano Ferreira, João Hugo Abdalla Santos, Geovana Ribeiro Pinheiro, Ligia Fernandes Abdalla, Lucas Carneiro dos Santos

**Affiliations:** ^1^ Hospital Adventista de Manaus, Manaus, Brazil;; ^2^ Universidade do Estado do Amazonas, Manaus, Brazil

## Abstract

This study presents a rare case of rhabdomyolysis caused by *Orthobunyavirus oropoucheense* in a 21-year-old male patient in the Amazon region. The diagnosis was confirmed by the reverse transcription polymerase chain reaction test. Neutropenia, thrombocytopenia, and hepatitis were also identified by laboratory tests, demonstrating the clinical relevance as they are alterations with potential severity. The patient’s good recovery is noteworthy, with only simple analgesia and intravenous hydration. The case highlights the need for better clarification regarding the pathophysiology of this distinct arbovirus.

## INTRODUCTION

Oropouche fever is an arboviral disease caused by *Orthobunyavirus oropoucheense* (OROV) belonging to the genus *Orthobunyavirus* and the family Peribunyaviridae.^1^ It is predominantly transmitted by the midge *Culicoides paraensis*, but other vectors, such as *Ochlerotatus serratus*, may also be involved.^2^^,^^3^ *Orthobunyavirus oropoucheense* was first isolated in Brazil in 1960 from the blood of a sloth (*Bradypus trydactilus*) during the construction of the Belém–Brasília highway and from mosquitoes captured in the same region. Since then, the virus has become an important pathogen in tropical and subtropical areas of Latin America.^4^

The epidemic potential of Oropouche fever was widely recognized in the 1970s when a major outbreak affected approximately 11,000 people. Since this initial outbreak, numerous others have been recorded in urban areas of the states of Acre, Amapá, Amazonas, Maranhão, Pará, Rondônia, and Tocantins.^5^ Despite its significant impact on public health in these regions, recognition of the disease remains limited, partly because of its clinical similarity to other arboviral infections, such as dengue virus (DENV), zika virus (ZIKV) and chikungunya virus (CHIKV).^6^

The diagnosis of Oropouche fever relies on specific laboratory methods. Detection of viral RNA by reverse transcription polymerase chain reaction (RT-PCR) is considered the gold standard for the acute phase, whereas serological tests, such as ELISA, can identify specific antibodies in later phases.^7^

Clinically, the disease presents fever; headache; myalgia; arthralgia; and other nonspecific symptoms, such as nausea and vomiting, in addition to cutaneous and hemorrhagic manifestations and in rare cases, complications of the central nervous system, including meningitis and encephalitis.^8^ Less than 1% of OROV cases progress to severe forms; however, accurately determining the mortality rate is challenging because of high underreporting and the lack of consistent laboratory diagnoses in endemic regions where the disease is recurrent.

A direct relationship between rhabdomyolysis and arboviral infections is not widely recognized as an established pattern. However, evidence suggests that some arboviruses, particularly in severe cases, may trigger this condition.^9^ Rhabdomyolysis is characterized by the breakdown of skeletal muscle tissue, leading to the release of intracellular components, such as myoglobin, which can impair renal function and progress to acute kidney injury.^10^

This study describes a rare case of rhabdomyolysis and hepatitis associated with Oropouche fever, and there are no previous publications in the literature. The clinical presentation and laboratory findings reveal potentially severe alterations, including hemorrhagic manifestations. This renders the case of significant scientific relevance as it supports the broad understanding of differential diagnoses and contributes to enhanced diagnostic approaches for this re-emerging arbovirus.

## CASE REPORT

We report the case of a 21-year-old male from Manaus, Amazonas with no known comorbidities or recent medication use who presented with chills and intense myalgia that started 1 day prior along with a documented fever of 38.1°C and arthralgia.

At admission, the patient was febrile and tachycardic (heart rate: 110 beats per minute) with a diffuse maculopapular rash localized to the thoracic region. Initial laboratory investigations revealed marked elevations in hepatic transaminases (alanine aminotransferase [ALT]: 230 U/L; aspartate aminotransferase [AST]: 749 U/L) and creatine phosphokinase (CPK; 41,650 U/L) (Table 1). Rapid diagnostic tests for NS1 antigen and *Plasmodium* spp. were negative.

**Table 1:** Laboratory tests

Day of Disease	Day 2	Day 3	Day 7	Day 13
Hemoglobin (g/dL)	15.2	13.7	15.3	15.7
Hematocrit (%)	42.5	39.7	43.2	45.4
Total leukocytes (*µ*L)	3,410	3,280	3,450	6,160
Neutrophils (*µ*L)	3,192	1,378	1,277	3,819
Lymphocytes (*µ*L)	546	1,476	1,587	1,786
Platelets (*µ*L)	206,000	133,000	124,000	305,000
C-reactive protein (mg/L)	12	29	6	5
AST (U/L)	749	1,000	345	83
ALT (U/L)	230	315	268	148
Urea (mg/dL)	18	6	15	19
Creatinine (mg/dL)	1.1	0.72	0.55	0.5
CPK (U/L)	41,650	53,590	3,848	527
Albumin (mg/dL)	–	–	4	5

ALT = alanine aminotransferase; AST = aspartate aminotransferase; CPK = creatine phosphokinase. Data were collected from the electronic medical record. Reference values are as follows: hemoglobin: 13.5–17.5 g/dL; hematocrit: 37–44%; leukocytes: 4,000–10,000 *µ*L; neutrophils: 1,500–8,000 *µ*L; lymphocytes: 900–4,000 *µ*L; platelets: 150,000–450,000 *µ*L; C-reactive protein: <5 mg/L; AST: up to 40 U/L; ALT: up to 56 U/L; urea: 15–45 mg/dL; creatinine: 0.7–1.3 mg/dL; CPK: 32–294 U/L; and albumin: 3.5–5 mg/dL.

**Figure 1.**
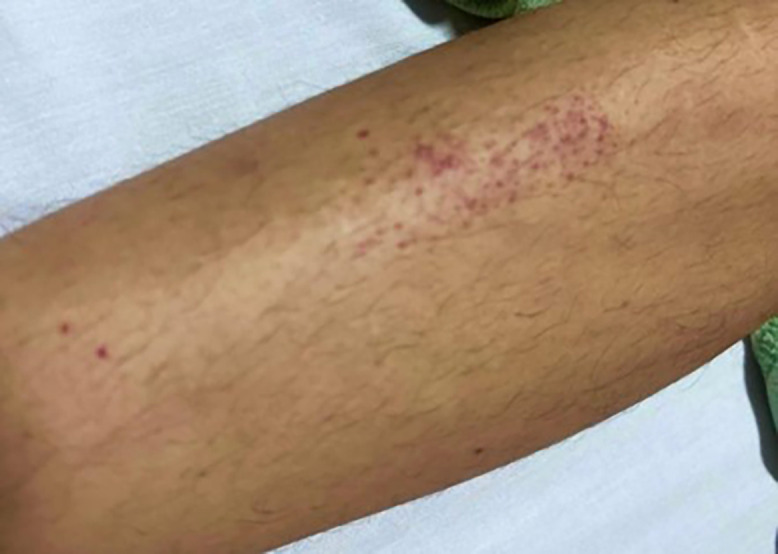
Clustering of multiple petechiae of small diameter (<2 mm) predominantly distributed in the anterolateral region of the leg, with an approximate extent of 5–7 cm. The lesions are nonconfluent and of an erythematous-purpuric color without signs of bruising, infiltration, or necrosis. The pattern and distribution are compatible with mild to moderate vasculitic or thrombocytopenic manifestations without visible severe hemorrhagic involvement.

On the first day of hospitalization, the patient remained hemodynamically stable, although myalgia and rash persisted. Petechial lesions subsequently developed on the chest and lower limbs (Figure 1). Physical examination revealed pain in the right hypochondrium; an abdominal ultrasound provide evidence of incidentally bilateral hydronephrosis.

After 48 hours of hospitalization, the patient became afebrile and reported partial clinical improvement, with only mild residual myalgia. However, follow-up laboratory results demonstrated worsening laboratory parameters, including a spike in CPK levels (53,590 U/L), elevated transaminases, thrombocytopenia, and leukopenia (Table 1). Management included intravenous saline hydration and symptomatic treatment with simple analgesics.

The diagnosis was confirmed through molecular testing performed on serum samples sent to the Leônidas and Maria Deane Institute, a unit of Fiocruz under the Brazilian Ministry of Health located in the state of Amazonas. The samples were analyzed by RT-qPCR and tested negative for mayaro virus, ZIKV, CHIKV and DENV.

Detection of OROV was positive (cycle threshold 32.22) and performed using the following primers and probe: direct primer (OROV_FNF): 5′-TCCGGAGGCAGCATATGTG-3′; reverse primer (OROV_FNR): 5′ACAACACCAGCATTGAGCACTT-3′; and probe (OROV_FNP): 5′(FAM) CATTTGAAGCTAGATACGG-3′.^11^

Over the following days, leukocyte and neutrophil counts progressively recovered, with leukopenia reaching 2,160/mm³ and neutropenia reaching 816/mm³ (Table 1). We conducted additional serologic tests, which were negative for hepatitis viruses, HIV, cytomegalovirus (CMV), and Epstein–Barr virus (EBV). Other differential diagnoses were not investigated because of low clinical suspicion.

Serologic results showed IgG positivity for CMV and EBV, suggesting past exposure. We also detected both IgM and IgG antibodies for dengue virus consistent with a previous infection, which the patient confirmed and the medical records corroborated.

**Figure 2.**
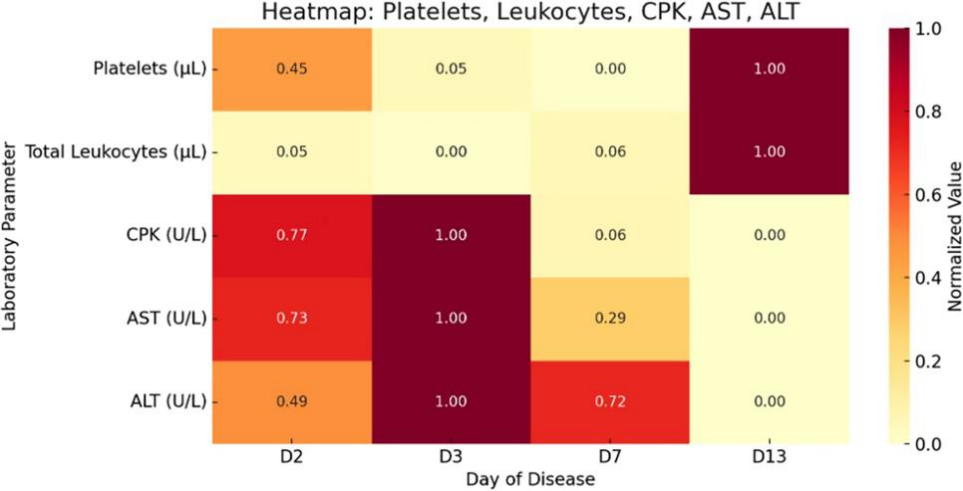
Dynamic changes in laboratory markers during illness. Data were collected from the electronic medical record. ALT = alanine aminotransferase; AST = aspartate aminotransferase; CPK = creatine phosphokinase.

After 7 days of hospitalization, we discharged the patient in clear clinical improvement and referred him for outpatient follow-up with infectious disease specialists. Laboratory tests showed normalization of leukocyte and platelet counts and a significant decline in CPK, AST, and ALT levels (Figure 2). By the 12th day after symptom onset, the patient remained asymptomatic, with all laboratory parameters within normal limits.

## DISCUSSION

In recent years, Oropouche fever has emerged as a significant public health challenge in Brazil, with a notable increase in reported cases in endemic regions and a geographic expansion beyond the Legal Amazon.^12^ In 2024, more than 6,600 cases were confirmed in the country, with most concentrated in the states of Amazonas (3,500 cases) and Rondônia (1,700 cases) but with notifications in all regions according to data from the Ministry of Health.^13^

In the last 70 years, at least 30 outbreaks of OROV have been recorded in Latin American countries, including Brazil, Peru, Colombia, French Guiana, and Panama, configuring an important emerging arbovirus in Latin America.^3^

Characteristic laboratory abnormalities, such as leukopenia and thrombocytopenia, are common in various arboviral infections, including Oropouche fever.^14^ Thrombocytopenia is usually observed around 4–5 days of illness; when associated with other coagulation alterations, it can be fatal.^15^ However, the neutropenia observed in this case, persisting for 4 days, is noteworthy as it is a hematological abnormality more frequently associated with the critical phase of dengue and rarely described in other arboviruses.^16^

Additionally, the tropism of the Oropouche virus for the liver, evidenced by hepatocyte necrosis and tissue lesions, has already been described, but clinically manifest hepatitis cases have not been widely reported.^17^ In this case, the significant elevation of hepatic transaminases, more than four times the upper limit of normal, suggests an infectious hepatitis associated with Oropouche fever, representing an uncommon and noteworthy manifestation.

Although rare in arbovirus infections, rhabdomyolysis can occur in severe cases and is most often associated with dengue and chikungunya.^9^ Studies in Brazil in 2017 estimate that 1.4% of patients with dengue may develop this complication.^18^ The pathophysiology of Oropouche fever, with viral replication in endothelial cells, leukocytes, and lymphocytes, promotes an acute inflammatory response that in severe cases, may lead to direct muscle damage and rhabdomyolysis.^17^

Rhabdomyolysis has several causes, including medications, toxins, infections, electrolyte imbalances, endocrinopathies, and inflammatory myopathies.^19^ Among bacterial infections, the most notable are legionella, tularemia, streptococcus, *Salmonella*, *Escherichia coli*, leptospirosis, *Coxiella burnetii* (Q fever), and staphylococcal infections.^20^

Viral myositis that progresses to rhabdomyolysis is uncommon but can be fatal. The viruses most associated with this complication are influenza A and B (the most common), Coxsackievirus, EBV, herpes simplex virus, parainfluenza virus, adenovirus, echovirus, CMV, measles virus, varicella-zoster virus, HIV, and currently, coronavirus disease 2019.^21^

In the case reported, high CPK values reinforce the diagnosis; however, persistent myalgia, concentrated urine, and asthenia are important to pay attention to as they help in the suspicion of rhabdomyolysis, thus avoiding progression to kidney injury, an unfavorable and fortunately, infrequent outcome in viral myositis.^22^^,^^23^ Our patient did not present symptoms of upper respiratory tract, jaundice, or gastrointestinal changes, which directed the search for relevant differential diagnoses.

It is important to emphasize that hemorrhagic manifestations, such as epistaxis, gingivorrhagia, and petechiae, are already described in up to 16% of Oropouche cases.^24^ Therefore, it is necessary to include it as a differential diagnosis in hemorrhagic febrile syndromes considering the emergence of the new recombinant OROV strain that is responsible for the recent outbreaks in Brazil.^3^

## CONCLUSION

This case illustrates the complexity of managing Oropouche fever, highlighting the importance of a comprehensive differential diagnosis and continuous monitoring in endemic regions. The rarity of manifestations, such as rhabdomyolysis and infectious hepatitis, underscores the need for further studies to better elucidate the pathogenesis and possible complications of this neglected arboviral disease.
